# Formaldehyde Induces Mesenteric Artery Relaxation via a Sensitive Transient Receptor Potential Ankyrin-1 (TRPA1) and Endothelium-Dependent Mechanism: Potential Role in Postprandial Hyperemia

**DOI:** 10.3389/fphys.2019.00277

**Published:** 2019-03-28

**Authors:** L. Jin, G. Jagatheesan, L. Guo, M. Nystoriak, M. Malovichko, P. Lorkiewicz, A. Bhatnagar, S. Srivastava, D. J. Conklin

**Affiliations:** ^1^Department of Anesthesiology, Critical Care and Pain Medicine, The Second Affiliated Hospital and Yuying Children’s Hospital of Wenzhou Medical University, Wenzhou, China; ^2^Department of Pharmacology and Toxicology, University of Louisville, Louisville, KY, United States; ^3^Envirome Institute, University of Louisville, Louisville, KY, United States; ^4^Diabetes and Obesity Center, University of Louisville, Louisville, KY, United States; ^5^Department of Medicine, University of Louisville, Louisville, KY, United States; ^6^American Heart Association Tobacco Regulation and Addiction Center, University of Louisville, Louisville, KY, United States

**Keywords:** aldehydes, EDRF, formalin, mesenteric artery, nitric oxide, transient receptor potential ankyrin-1

## Abstract

Formaldehyde (FA), the smallest aldehyde, is generated endogenously, and is widespread in the environment in foods, beverages and as a gas phase product of incomplete combustion. The main metabolite of FA, formate, was increased significantly in murine urine (∼3×) after overnight feeding. Because feeding increases mesenteric blood flow, we explored the direct effects of FA in isolated murine superior mesenteric artery (SMA). Over the concentration range of 30–1,200 μM, FA strongly and reversibly relaxed contractions of SMA induced by three different agonists: phenylephrine (PE), thromboxane A_2_ analog (U46,619) and high potassium (60K, 60 mM K^+^). Formate (to 1.5 mM) induced a modest relaxation. FA (>1,500 μM) irreversibly depressed vascular function in SMA indicating vasotoxicity. The sensitivity (EC_50_) but not the efficacy (% relaxation) of FA-induced relaxations was dependent on blood vessel type (SMA << aorta) and contractile agonist (PE, EC_50_= 52 ± 14 μM; U46,619, EC_50_= 514 ± 129 μM; 60K, EC_50_= 1,093 ± 87 μM). The most sensitive component of FA vasorelaxation was within physiological levels (30–150 μM) and was inhibited significantly by: (1) mechanically impaired endothelium; (2) Nω-Nitro-L-arginine methyl ester hydrochloride (L-NAME); (3) transient receptor potential ankyrin-1 (TRPA1) antagonist (A967079); (4) guanylyl cyclase (GC) inhibitor (ODQ); and, (5) K^+^ channel inhibitor (BaCl_2_). A similar mechanism of SMA vasorelaxation was stimulated by the TRPA1 agonist cinnamaldehyde. Positive TRPA1 immunofluorescent staining and gene-specific sequence were present in SMA but not in aorta. These data indicate FA, but not formate, robustly relaxes SMA via a sensitive TRPA1- and endothelium-dependent mechanism that is absent in aorta. Thus, as FA levels increase with feeding, FA likely contributes to the physiological reflex of post-prandial hyperemia via SMA vasodilatation.

## Introduction

Formaldehyde (FA) is the simplest aldehyde and the concentration of FA in the blood ranges from 0.0127 to 2.28 μg/mL (1.9 to 5.4 mg/L; or 63–179 μM) (Wang et al., 2011; Kleinnijenhuis et al., 2013). FA can be found in food and beverages (likely the largest quantitative source of exogenous FA) and is metabolically derived (see Conklin et al., 2011). Exogenous FA is a volatile indoor and outdoor pollutant emitting from building materials (e.g., plywood, glues, carpet, drapery), and present in cosmetics, vehicle exhaust, photochemical smog and tobacco-derived aerosols (Conklin et al., 2011; Ogunwale et al., 2017). Endogenous FA is generated by the breakdown of L-methionine, histamine, methanol and methylamine, and may contribute to biological methylation of folic acid (Yu and Zuo, 1996). Thus, blood levels of FA are in constant flux due to rapid changes in exposure, absorption, distribution, metabolism (to formate and CO_2_) and excretion.

Although direct effects of FA on cardiovascular targets are described, the role of FA in cardiovascular physiology is not well explored. For example, intra-arterial or intravenous FA reduces heart rate (bradycardia), decreases arterial pressure (hypotension) and causes arrhythmias in animals (Tani et al., 1978, 1986; Strubelt et al., 1990; Tani and Horiguchi, 1990), but these are considered toxicological effects due to high doses administered directly into the blood. Because FA is toxic in cultured endothelial cells (inhibition of mitosis, increased apoptosis), vascular smooth muscle cells and cardiomyocytes (Conklin et al., 1998), it is, thus, speculated that endogenously generated FA induces endothelium dysfunction by which it accelerates diabetic complications such as atherosclerosis (Deng and Yu, 1999). However, FA induces a concentration-dependent relaxation in isolated blood vessels (Tani, 1981; Conklin et al., 2004; Zhang et al., 2018) yet the relevance of this effect in vascular physiology is unclear.

Despite myriad sources of FA and its provoked cardiovascular effects, few studies address the vascular effects of physiological levels of FA. Formate is a stable metabolite of FA generated from a 2-step enzymatic process involving glutathione-dependent formaldehyde dehydrogenase (FALDH2) hemi-thioacetal formation and aldehyde dehydrogenase 2 (ALDH2) mediated oxidation (Conklin et al., 2007). In the current study, we measured urine levels of formate, the primary metabolite of FA, to estimate changes in endogenous FA levels. As murine urine levels of formate increased ∼3 times overnight (with feeding) compared with levels after daytime fast, we postulate that feeding increases endogenous FA levels (directly or indirectly). As such, the gastrointestinal vasculature represents a likely target of changes in FA levels following feeding when reflexively blood flow increases in the gut (Takagi et al., 1988). Thus, to address potential FA-induced vascular effects, isolated superior mesenteric artery (SMA) and aorta were exposed to FA across a physiologic to toxic range (30–1,500 μM). Although an FA-induced vasorelaxation was present in both blood vessels, the most sensitive relaxation was present in SMA indicating that physiologic levels of FA likely stimulate dilation in SMA before aorta. Thus, we identified the mechanisms of the FA-induced relaxation (30–150 μM) as dependent on: a functional endothelium, TRPA1 activation, NO formation, guanylyl cyclase activation, and potassium channels. Formate, though, had minimal effect in blood vessels up to 2,100 μM. These data collectively support the idea that feeding increases FA levels, and that FA (and not formate) likely contributes to increased gastrointestinal blood flow of feeding (i.e., postprandial hyperemia) through a sensitive, robust and reversible TRPA1- and endothelium-dependent mechanism.

## Materials and Methods

### Chemicals and Solutions

Reagent grade chemicals were purchased from Sigma-Aldrich or commercial sources as indicated: A967079 (AdooQ); acetylcholine chloride (ACh); 4-aminopyridine (4-AP); barium chloride (BaCl_2_); cinnamaldehyde (≥95%); formalin (formaldehyde, 37% and methanol, 10–15%); formate; 1 h-[1,2,4]oxadiazolo[4,3-a]quinoxalin-1-one (ODQ); N^ω^-nitro-_L_-arginine methyl ester hydrochloride (L-NAME); L-phenylephrine hydrochloride (PE); sodium nitroprusside (SNP); tetraethylammonium chloride (TEA); and, U46,619 (thromboxane A_2_ analog). Krebs physiological salt solution (PSS) for SMA was (in mM): NaCl, 119; KCl, 4.7; CaCl_2_, 2.5; MgCl_2_, 1.2; KH_2_PO_4_, 1.2; NaHCO_3_, 24; glucose, 7.0; pH 7.4. Krebs PSS for aorta was (in mM): NaCl, 118; KCl, 4.7; CaCl_2_, 2.5; KH_2_PO_4_, 1.2; MgSO_4_, 1.2; NaHCO_3_, 12.5; and, glucose, 5.5; pH 7.4. High K^+^ PSS (60K; 60 mM) substituted equimolar K^+^ for Na^+^.

### Animals

Wild type C57BL/6J mice from in house breeding pairs or purchased (The Jackson Laboratory, Bar Harbor, ME, United States) (12–20 weeks old; 25–35 g) were used in these studies. Mice were treated according to American Physiological Society *Guiding Principles in the Care and Use of Animals*, and all protocols were approved by University of Louisville Institutional Animal Care and Use Committee. Mice were housed under pathogen-free conditions in the University of Louisville vivarium under controlled temperature and 12 h light: 12 h dark cycle. Mice were provided a standard chow diet (Rodent Diet 5010, 4.5% fat by weight, LabDiet; St. Louis, MO, United States).

### Urine Collection and Formate Measurement

Urine samples were collected, centrifuged (2,500 RPM, 10 min, 4°C), decanted, and stored at –80**°**C until analysis (Conklin et al., 2017). Each mouse was briefly and manually (<5 s) exposed on the mouth with a water bottle filled with D-glucose/saccharin solution (w/v; 3.0%/0.125%; Sigma) immediately prior to a 6 h fast. After fasting, each mouse was placed in a metabolic cage (Harvard Apparatus) with *ab libitum* access to glucose/saccharin solution in water without access to food for 3 h to collect urine (in 4**°**C water-jacketed organ baths). In total, mice were fasted for 9-h. After fasted urine collection, mice were provided food and glucose/saccharin solution *ab libitum* for overnight urine collection (O/N). Urine samples were collected, centrifuged, decanted, and stored at –80**°**C (Conklin et al., 2017).

The primary urinary metabolite of FA is formate and it was measured using GC-MS as described previously (Kage et al., 2004; Lamarre et al., 2014) with minor modifications. Briefly, urine (50 μL) was mixed with sodium phosphate (20 μL 0.5 M, pH 8.0) containing internal standard (^13^C-formate, 2.3 mM). Samples were incubated with PFBBr (130 μL, 0.1 M) for 15 min at 60°C, extracted with hexane (330 μL), and analyzed by GC-MS in electron ionization (EI) mode. Six-point calibration curve was used to calculate the concentration of formate. Results were corrected for the natural abundance of ^13^C isotope. Formate level was normalized to urinary creatinine (mg) to account for urine dilution.

### Isolated Superior Mesenteric Artery (SMA) and Aorta

Mice were anesthetized with sodium pentobarbital (0.1 ml, 150 mg/kg, i.p.), and SMA and aorta were removed via mid-ventral thoracotomy. Thoracic aorta rings (3–4 mm) were hung on stainless steel hooks in 15-ml water-jacketed organ baths and SMA rings (2 mm) were hung on tungsten wire (75 μm dia.) in 5-ml heated organ baths (MultiWire Myograph System 620M, DMT, Denmark) in PSS bubbled with 95% O_2_: 5% CO_2_ at 37°C. After 10 min without tension, aorta rings were equilibrated to ∼1 g loading tension over 30 min and SMA rings were equilibrated to ∼0.25 g loading tension over 1 h. All rings were stimulated with 60K to test for viability, washed 3 times with PSS over 30 min, and re-equilibrated to appropriate resting tension.

### FA-Induced Relaxation in Isolated Superior Mesenteric Artery (SMA) and Aorta

Formaldehyde (30–1,500 μM) was added to organ baths containing either aorta or SMAs pre-contracted with 1 of 3 contractile agonists: phenylephrine (PE, 10 μM), U46,619 (0.1 μM), or 60K (60 mM). The efficacy of FA-induced relaxation was calculated as the % reduction in agonist-induced contraction. The sensitivity of FA-induced relaxation was calculated as the effective concentration producing 50% response (EC_50_), i.e., cumulative concentration responses were normalized to 100% with interpolation of EC_50_. To assess whether time of day had an effect on SMA response to FA, mice were euthanized both in day and in night cycle. The night-time mice were removed from an animal housing room with a switched 12 h:12 h dark:light cycle after 1 week acclimation prior to euthanization.

### Effects of Formate (FA Metabolite) and Methanol

Formaldehyde is oxidized to formate (primary urinary metabolite), and thus, we tested whether formate could induce vasorelaxation or vascular toxicity (50 to 1,500 μM) in PE pre-contracted aorta and SMA. Because formalin (a source of FA) contains 10–15% methanol (as a stabilizer), we also tested whether formalin or methanol alone (to 0.001%, equivalent to amount present in 1,200 μM formalin) contributed, in part, to FA-induced relaxation.

### Role of Endothelium, Nitric Oxide Synthase (NOS), and cGMP

To evaluate the role of NOS, L-NAME (0.1 mM) was added prior (15 min) to addition of PE (10 μM), U46,619 (0.1 μM) or High K^+^ (60 mM). Pre-contracted SMA rings were then relaxed with cumulative concentrations of FA (50, 150, and 300 μM). The role of the endothelium was assessed as vasorelaxation to increasing FA concentrations (50, 150, 300, and 600 μM) in aorta and in SMA with intact and injured endothelium. The endothelium was mechanically injured by air perfusion, and effective impairment was confirmed by near complete abolition (>95%) of ACh-induced dilation of PE pre-contracted blood vessels. To assess whether cGMP was involved in FA-induced vasorelaxation, isolated blood vessels were pre-incubated with ODQ (3 μM) to inhibit guanylyl cyclase (GC) and subsequent formation of cGMP (Jiang et al., 2015).

### Role of TRPA1 Channel

The role of the TRPA1 channel was assessed by addition of TRPA1 antagonist (A967079, 1 μM; 10 min; Cat. #: A12353, AdooQ, Invine, CA, United States) or DMSO (vehicle) in PE-precontracted aorta and SMA followed by cumulative addition of FA (30–300 μM). Sensitivity of SMA to a known TRPA1 agonist, cinnamaldehyde (1–100 μM), was tested. Because cinnamaldehyde is immiscible in aqueous solution, dilutions were vigorously vortexed immediately prior to addition to organ baths.

### Role of K^+^ Channels

The role of K^+^-channels in FA-induced responses were interrogated using K_IR_ channel inhibitor (BaCl_2_, 1 mM) and the non-specific Kv channel inhibitor (tetraethylammonium, TEA, 10 mM).

### Histology and TRPA1 Immunofluorescence

Thin sections (4 μm) of formalin-fixed, paraffin-embedded SMA and dorsal root ganglia (DRG; positive control) of WT mice were stained with H&E and TRPA1 antibody (1:200; Alomone Labs, Israel; Cat. #: ACC-037). Images of SMA sections were made using a digital Spot camera mounted on an Olympus microscope. Immunofluorescence microscopy was performed with rabbit polyclonal antibody against TRPA1 without or with a blocking peptide of the TRPA1 antibody and Alexa Fluor 647 goat anti-rabbit secondary antibody (1:400 dilution; Invitrogen; Cat. #: 21244). To label endothelial cells, sections were co-stained with isolectin GS-IB4 Alexa Fluor 594 conjugate (1:200 dilution; Invitrogen; Cat. #: I21413). Briefly, the slides were deparaffinized, and heat-mediated antigen retrieval was performed in 10 mM citrate buffer (pH 6.0) for 20 min at 95°C. The slides were cooled to room temperature and then incubated with blocking buffer (10% goat serum, 1% BSA, 0.025% triton X-100 in 1× TBS) for 2 h at RT and then incubated with either TRPA antibody in blocking buffer without and with blocking peptide against TRPA1 (the TRPA1 antibody and the blocking peptide at 1:1 ratio; μg/μg) was mixed and pre-incubated for 1 h at RT before adding on top of the tissue sections) in blocking buffer overnight at 4°C. The slides were incubated with anti-rabbit Alexa Fluor 647 secondary antibody in the dark for 1 h at RT. The slides were washed 3 × 5 min with 1× TBST after each step. Tissue sections incubated with secondary antibody only served as a negative control. Slides were covered with DAPI containing Slow Fade^®^ Gold anti-fade reagent (Invitrogen; Cat. #: S36938) and fluorescence was visualized on a Nikon eclipse Ti fluorescence Microscope using NIS-Elements (Nikon; Japan) at 200×. A DAPI filter was used for nuclear staining and a Cy5 Red filter for TRPA1 staining.

### TRPA1 qRT-PCR

Total RNA was isolated from approximately 10 mg of SMA (8 arteries pooled) and DRG (positive control) from WT mice with Trizol (Invitrogen) and RNeasy MiniKit (QIAGEN) according to manufacturer’s instructions. RNase-free DNase (QIAGEN; Cat. # 79254) was used to remove any contaminating DNA. RNA was eluted from RNeasy mini-columns into 30 μL of RNAse-free water and the concentration measured using NANODROP 2000C (Thermo Fisher Scientific). Total cDNA was synthesized using iScript cDNA synthesis Kit (BIO-RAD; Cat. # 170-8891) according to manufacturer’s instructions. Briefly, 15 μL denatured RNA (250 ng), 4 μL 5× iScript reaction mix and 1 μL of iScript reverse transcriptase were mixed (total volume, 20 μL) and cDNA synthesis was carried out in a Bio-Rad mycycler Thermocycler using the following conditions: 42**°**C for 60 min and 94**°**C for 5 min. The resultant cDNA was target amplified using TRPA1 specific primers. (Forward 5′-*GAT GCC TTC AGC ACC CCA TTG-*3′; Reverse 5′-*CAC TTT GCG TAA GTA CCA GAG TGG*-3′). Real-time quantitative PCR was done using a murine specific TaqMan gene expression assay. Briefly, the following were added to each well of a 384-well plate sequentially: 5.0 μL of 2× TaqMan Fast Universal PCR Master Mix (Cat.no. 4440040), 2 μL undiluted cDNA from target specific PCR and 3 μL of murine primers for *mTrpa1* (Mm01227447_m1, Cat. # 4351372) or *β*-*actin* (Mm00607939_m1, Applied Biosystems, Cat. # 4331182) for TaqMan assays (total = 10 μL final volume), and then subjected to qRT-PCR using standard protocols on an Applied Biosystems 7900 HT Real-Time PCR system. For each RNA sample, the cDNAs were run in duplicate for each assay set in the same plate along with β-actin. The expression level of TRPA1 in both SMA and DRG are expressed as ct values.

### DNA Sequencing and Agarose Gel Electrophoresis

PCR products from TRPA1 TaqMan assay were run on 1.5% agarose gel and stained with ethidium bromide to visualize the expected 101 bp band in WT SMA and DRG. Also, the PCR product from target specific TRPA1 amplification (309 bp) of SMA and DRG were sequenced on Illumina NextSeq 500 by the Center for Genetic and Molecular Medicine (CGEMM University of Louisville) sequencing service core.

### Statistical Analyses

Data are expressed as means ± SE. When comparing two groups, a *t*-test was used (paired or unpaired as appropriate), and statistical significance was assumed where *P* < 0.05 (SigmaPlot, ver. 11).

## Results

### Formate Is Increased With Feeding

Formate is the primary metabolite of FA, and formate in the urine is a consequence of endogenous FA metabolism, so we measured urinary formate by GC-MS. We found that feeding significantly increased the urinary levels of formate indicating a major source of endogenous FA is either directly from food or related to feeding, e.g., metabolism of food constituents, intermediary metabolism, etc. In male C57BL/6J mice, a daytime fast (9 h) resulted in a basal level of urinary formate that was tripled with overnight feeding (Table 1). A similar pattern occurred in female C57BL/6J mice (data not shown). Thus, we infer that feeding increased FA levels that subsequently increased formate excretion in urine.

**Table 1:** Levels of urinary formate following either a daytime fast or overnight feeding in male C57BL/6J mice.

	Condition	
Urinary Metabolite	Daytime Fast	Overnight Feeding
Formate (μg/ml)	0.72 ± 0.03	2.73 ± 0.95^∗^
Creatinine (mg/dl)	6.88 ± 0.32	7.23 ± 0.66
Formate (μg/mg creatinine)	10.54 ± 0.74	36.82 ± 10.30^∗^

*Values = means ± SE. *N* = 3–5 per group. ^∗^, *P* < 0.05 vs. fasting.*

### FA-Induced Relaxation

**FIGURE 1:**
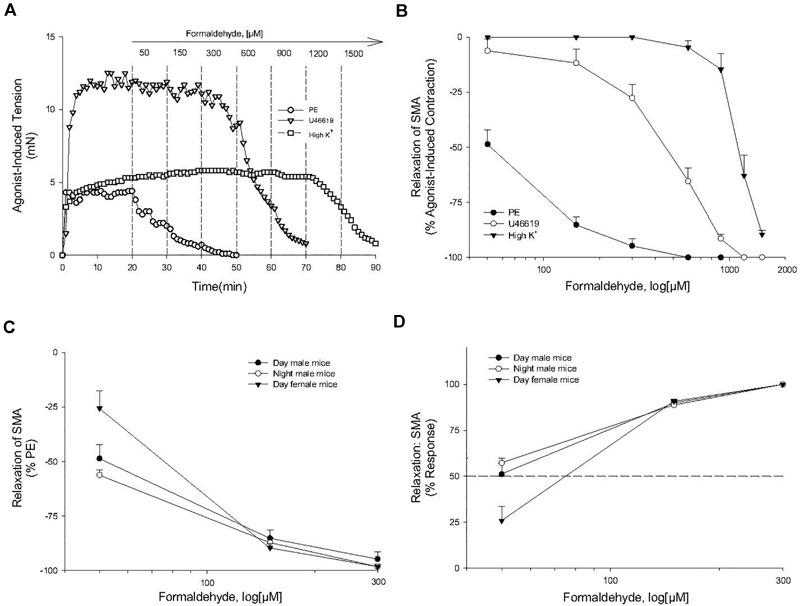
Formaldehyde (FA) induced relaxation in superior mesenteric artery (SMA). **(A)** Representative traces of concentration-dependent relaxation induced by cumulative addition of FA (50–1,200 μM) in isolated SMA contracted by one of three different contractile agonists: phenylephrine (PE); thromboxane A_2_ analog (U46,619); or, high potassium (60K). **(B)** Summary data of the efficacy (% relaxation) of FA-induced relaxation in isolated pre-contracted SMA. **(C,D)** Summary data of the efficacy (% relaxation; **C**) and of the sensitivity **(D)** of FA-induced relaxation in isolated PE-precontracted SMA from male and female mice isolated either during day time or night time. Values are means ± SE of 3–10 preparations.

Formaldehyde (from 30 μM to 1.2 mM) induced a robust, repeatable and concentration- and agonist-dependent relaxation in isolated SMA and aorta of both male and female mice (Figure 1A–D). Because blood vessels of male and female mice removed during light (day) or dark (night) cycle were equally sensitive to FA, subsequent experiments were done with blood vessels of male mice in light cycle (Table 2). Regardless of pre-contraction agonist (PE, U46,619, 60K), FA relaxed agonist-induced tension by >90% in SMA and aorta (Table 3), yet when added to an uncontracted vessel, FA did not affect basal tone (myogenic tone; data not shown). FA-induced relaxation (up to 1.2 mM in SMA; and up to 1.5 mM in aorta) did not alter subsequent agonist-induced contractions or ACh-induced (endothelium-dependent) relaxation indicating no overt vasotoxicity. Irreversible toxicity was, however, detected following exposure to high levels of FA (>1.2 mM in SMA; >1.5 mM in aorta) with vasotoxicity defined as the near loss of contractility even after multiple bath changes (e.g., 3 washouts, <10% tension production of first agonist-induced contraction; data not shown).

**Table 2:** The effective concentration inducing 50% relaxation (EC_50_; in μM) of formaldehyde-induced vasorelaxation in isolated murine superior mesenteric artery (SMA; male and female; day and night) and thoracic aorta precontracted with different agonists.

Blood Vessel (sex, time of day)	EC_50_ by precontraction agonist		
	PE	U46,619	60K
SMA (male, day)	53 ± 3	514 ± 129^∗^	1,093 ± 87^∗^
SMA (male, night)	43 ± 2	–	–
SMA (female, day)	77 ± 9	–	–
Aorta (male, day)	261 ± 22^∗^	–	–

*Values = means ± SE. Abbr.: PE, phenylephrine, [10 μM]; U46,619, thromboxane A_*2*_ analog, [0.1 μM]; 60K, high potassium solution, [60 mM K^+^]; –, did not perform experiment. *N* = 3–10 per group. ^∗^, *P* < 0.05 vs. EC_*50*_ of “SMA (male, day)” PE agonist (i.e., baseline control).*

**Table 3:** Efficacy of formaldehyde and associated compounds in relaxation of PE-precontracted superior mesenteric artery (SMA) and aorta without and with selective treatments (inhibitors).

Compound	[mM]		Treatment	% Relaxation	
	SMA	Aorta		SMA	Aorta
Formaldehyde	0.3	0.6	–	-94.3 ± 1.3	-99.6 ± 1.2
Formate	2.1	–	–	-25.7 ± 6.7	–
Methanol	2.1	–	–	-34.1 ± 11.7	–
Formaldehyde	0.3	0.6	ED	-90.4 ± 2.3	-94.0 ± 2.6
Formaldehyde	0.6	–	+LNAME	-96.7 ± 2.2	–
Formaldehyde	0.3	0.6	+A967079	-96.4 ± 1.1	-100.7 ± 1.2
Formaldehyde	0.6	–	+ODQ	-99.7 ± 0.5	–
Formaldehyde	0.6	0.6	+BaCl_2_	-99.5 ± 0.5	-81.7 ± 3.7
Formaldehyde	0.2	0.6	+TEA	-90 ± 1.8	-94.4 ± 1.3

*Values = means ± SE. Abbr.: PE, phenylephrine, 10 μM; ED, endothelial dysfunction induced by mechanical air perfusion; LNAME, N^ω^-nitro-_*L*_-arginine methyl ester hydrochloride, 100 μM; A967079, TRPA1 antagonist, 1 μM; 3 μM; ODQ, 1 h-[1,2,4]oxadiazolo[4,3-a]quinoxalin-1-one, BaCl_*2*_, barium chloride, 1 mM; TEA, tetraethylammonium. *N* = 3–6 per group.*

### FA: Formate and Methanol

Because FA is metabolized to formate, it is possible that vascular effects of FA (and its precursors) are the result of FA metabolism to formate. To address this possibility, formate was cumulatively added up to 2.1 mM in PE pre-contracted SMA. Formate induced a weak relaxation indicating that FA-induced relaxation was not a result of formate production (Table 3). Formalin, a mixture of FA (37%) and methanol (10–15% as stabilizer), induces TRPA1-dependent vasodilation and pain (McNamara et al., 2007), yet it is unknown if this is due to either FA or methanol (or both). Because FA also is a product of methanol metabolism, we tested whether methanol alone (at levels comparable with those present in formalin) had any effect in PE pre-contracted SMA. Methanol also induced a weak, modestly concentration-dependent relaxation in SMA. Thus, neither formate nor methanol appears responsible for the efficacy of FA- or formalin-induced relaxations, respectively. These data also implicate real time metabolism of FA (to formate) as a relevant vaso-regulatory step because it converts an active agent (FA) into an inactive metabolite (formate). Thus, alterations in FA metabolism in the vascular wall may be a targetable process to promote or diminish feeding-induced hyperemia.

### Role of Endothelium, Nitric Oxide Synthase (NOS), and GC in FA-Induced Vasorelaxation

**FIGURE 2:**
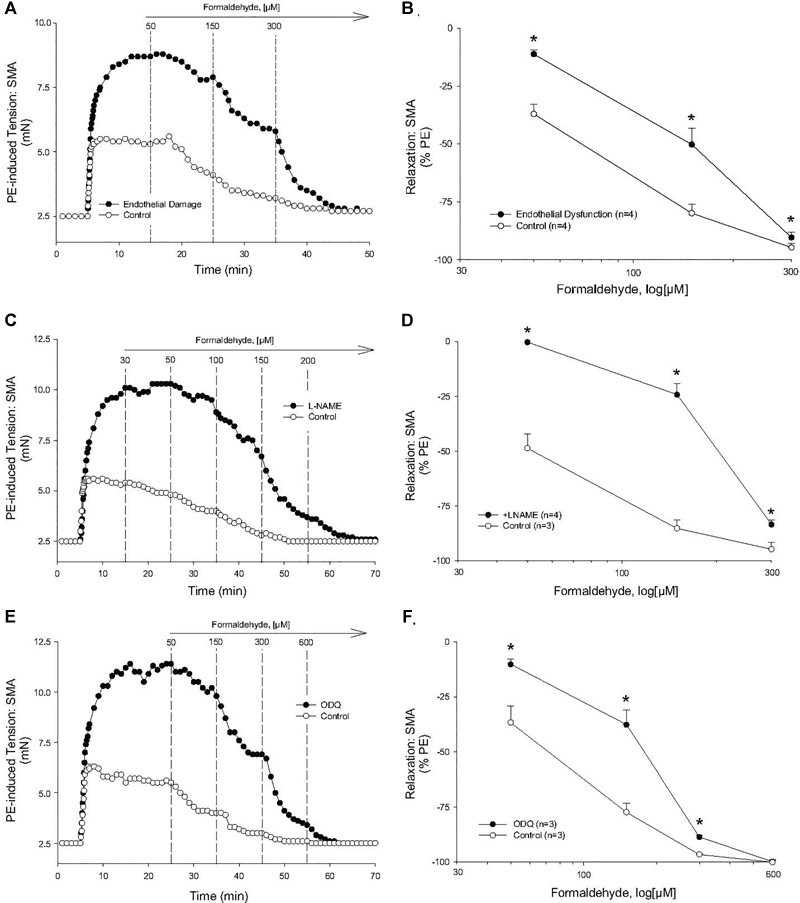
Role of the endothelium and nitric oxide (NO) in the most sensitive component of formaldehyde-induced relaxation of phenylephrine (PE) pre-contracted SMA. **(A)** Representative traces of FA-stimulated relaxation of PE-induced contraction without and with endothelium impairment due to 5 min of air perfusion. **(B)** Summary data of FA-induced relaxation in isolated SMA pre-contracted with PE in the presence and absence of functional endothelium. **(C)** Representative traces of FA-stimulated relaxation of PE-induced contraction in the presence and absence of LNAME. **(D)** Summary data of FA-induced relaxation in isolated SMA pre-contracted with PE in the absence and presence of LNAME. **(E)** Representative traces of FA-stimulated relaxation of PE-induced contraction in the presence and absence of the guanylyl cyclase (GC) antagonist, ODQ (3 μM), added to bath after PE-induced contraction plateaued and prior to cumulative addition of FA. **(F)** Summary data of FA-induced relaxation in isolated SMA pre-contracted with PE in the absence and presence of ODQ. Values are means ± SE of 3–4 preparations. ^∗^, *P* < 0.05 vs. Control.

To investigate the mechanism of the most sensitive component of the FA-induced relaxation, we used PE-precontracted SMA (EC_50:_ PE < U46,619 < 60K). Because the SMA has a robust ACh-induced, endothelium-dependent relaxation that was impaired significantly by air perfusion (Supplementary Figure S1A), we tested for the role of endothelium in FA-induced relaxation. Air perfusion significantly shifted FA-induced relaxation rightward in SMA (Figure 2A,B). Similarly, the sensitivity (EC_50_) but not the efficacy of FA-induced vasorelaxation in PE-pre-contracted SMA was affected by LNAME (100 μM) (Figure 2C,D). However, LNAME had no effect on sensitivity or efficacy of FA-induced relaxation in either U46,619 (EC_50_: 514 ± 129 vs. 569 ± 98 μM) or 60K (EC_50_: 1,093 ± 87 vs. 984 ± 92 μM) pre-contracted SMA (Supplementary Figures S1C,D). The rightward shift due to LNAME indicated a role of NO (e.g., eNOS) as part of the most sensitive component of the FA-induced relaxation in PE-precontracted SMA. Because NO stimulates guanylyl cyclase (GC) to generate cGMP in smooth muscle cells, we tested ODQ, an irreversible inhibitor of GC, and ODQ also rightward shifted the FA-induced relaxation (Figure 2E,F). The endothelium contributes to overall contractility in SMA (Supplementary Figures S1B,E). Collectively, these data show that *the most sensitive component of the FA-induced relaxation is dependent on a functional endothelium, NOS and an NO-mediated activation of GC*.

### Role of TRPA1 Channel

**FIGURE 3:**
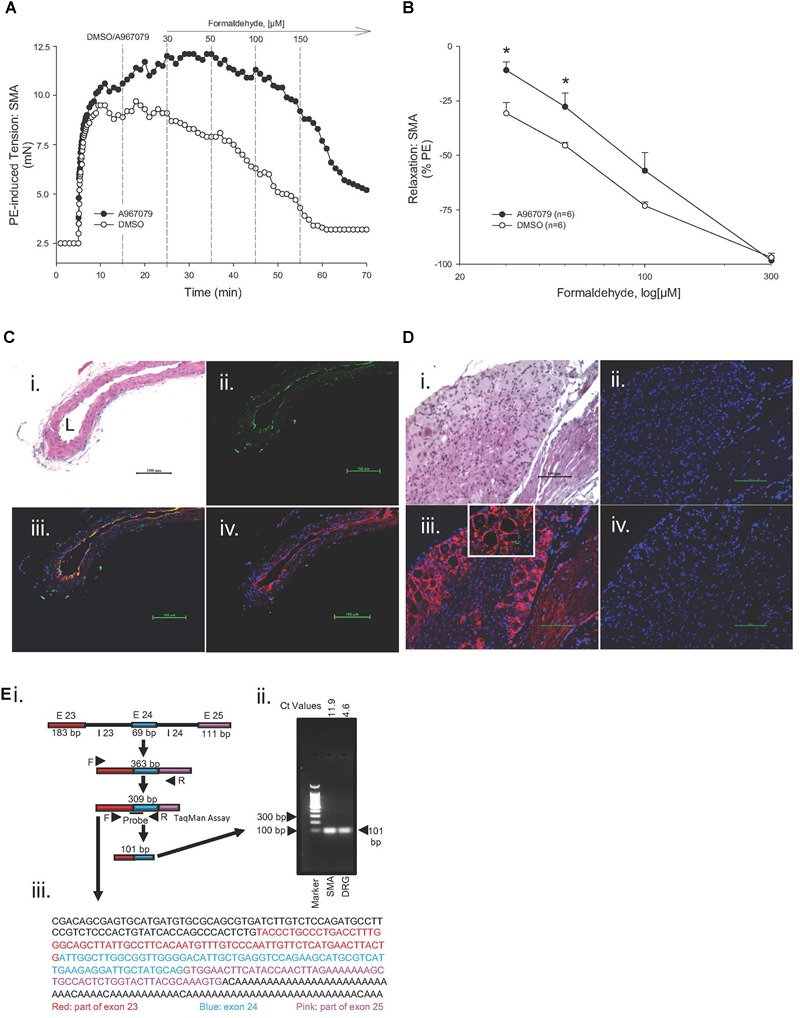
Role of the TRPA1 channel in formaldehyde (FA)-induced relaxation in phenylephrine (PE) pre-contracted SMA. **(A)** Representative traces of FA-stimulated relaxation of PE pre-contracted SMA in the absence (Control) and presence of the TRPA1 antagonist, A967079 (3 μM), which was added after PE-induced contraction plateaued and prior to cumulative addition of FA in wild type (WT) SMA. **(B)** Summary data of FA-induced relaxation in isolated SMA pre-contracted with PE in the absence and presence of TRPA1 antagonist. Immunofluorescence localization of TRPA1 in SMA **(C)** and in dorsal root ganglion (DRG; **D**). Formalin-fixed and paraffin-embedded sections of murine organs were stained with H&E **(Ci, Di)**; TRPA1 only (green; **Cii**) or DAPI only (blue, nuclear stain; **Dii**); TRPA1 antibody (green), isolectin (red, endothelium) and DAPI **(Ciii)** or TRPA1 antibody (red) and DAPI **(Diii)**; and, TRPA1 antibody, TRPA1 blocking peptide, isolectin and DAPI **(Civ)** or TRPA1 antibody, TRPA1 blocking peptide, and DAPI **(Div).** L, lumen of SMA. All images were at 200× magnification (scale bar = 100 μm). **Ei**) Schematic depicting mouse *mTrpa1* gene exons **(E)** 23 to 25 (with introns, I) and their length (in base pairs, bp). TRPA1 mRNA with the spliced exons 23 to 25 makes a 309 bp product as indicated by TRPA1 specific primers used. **(Eii)** Agarose gel electrophoresis of PCR amplified product from 309 bp cDNA by qRT-PCR (101 bp product from SMA or DRG of WT mouse using TaqMan Assay for TRPA1; ct values indicated). **(Eiii)** DNA sequence of 309 bp PCR product identified as TRPA1. Sequence of DRG PCR product (309 bp) was used as validation (data not shown). Values are means ± SE of 3–4 preparations. ^∗^, *P* < 0.05 vs. Control.

Because formalin-induced pain is associated with activation of TRPA1 channel as well as an increase in blood flow (McNamara et al., 2007), we hypothesized that the TRPA1 channel may contribute to the FA-induced, endothelium-dependent relaxation in SMA. In support, the TRPA1 antagonist A967079 (1 μM) significantly inhibited the most sensitive portion of the FA-induced relaxation (30 μM) in PE-contracted SMA (Figure 3A,B). In paraffin-embedded, thin cut sections of mouse SMA, TRPA1 (green) was co-localized with isolectin (red) in endothelium (Figure 3C). More modest yet specific TRPA1 staining was present in media and adventitia of SMA as well. Positive and selective TRPA1 staining was confirmed by inclusion of a blocking peptide, and by using dorsal root ganglion (DRG; Figure 3D) as a positive control. A PCR product specific to TRPA1 exons 23–25 mRNA (Figure 3Ei) matched both the predicted fragment size (101 bp; Figure 3Eii) and by gene sequence (Figure 3Eiii) – confirming TRPA1 presence in SMA. These image and molecular data support that TRPA1 contributed to the FA-induced, endothelium-dependent relaxation in SMA.

**FIGURE 4:**
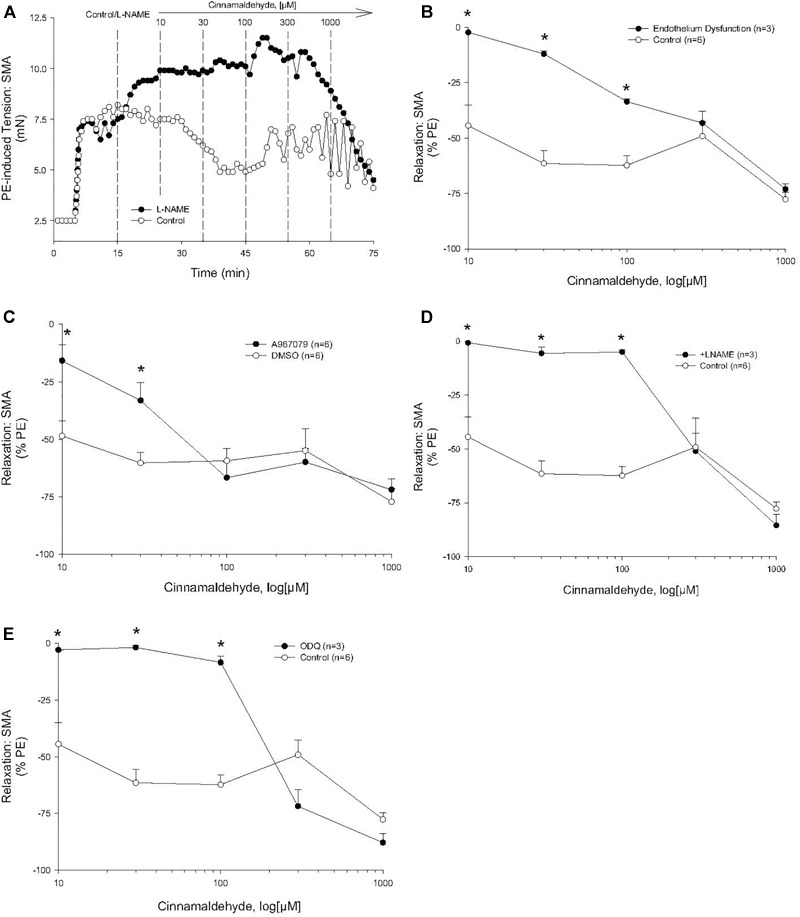
Role of the endothelium and nitric oxide (NO) in the most sensitive component of cinnamaldehyde-induced (CA) relaxation of phenylephrine (PE) pre-contracted SMA. **(A)** Representative traces of concentration-dependent CA-stimulated relaxation of PE-induced contraction. **(B)** Summary data of CA-induced relaxation in isolated SMA pre-contracted with PE in the presence and absence of functional endothelium. **(C)** Summary data of CA-induced relaxation in isolated SMA pre-contracted with PE in the absence and presence of TRPA1 antagonist. **(D)** Summary data of CA-induced relaxation in isolated SMA pre-contracted with PE in the absence and presence of LNAME. **(E)** Summary data of CA-stimulated relaxation of PE-induced contraction in the presence and absence of the guanylyl cyclase (GC) antagonist, ODQ (3 μM), added to bath after PE-induced contraction plateaued and prior to cumulative addition of CA. Values are means ± SE of 3–4 preparations. ^∗^, *P* < 0.05 vs. Control.

Cinnamaldehyde is a known TRPA1 agonist, and, unsurprisingly, it induced a concentration- and TRPA1-dependent relaxation in SMA (Figure 4A). Like FA, this sensitive cinnamaldehyde-induced relaxation in SMA was dependent on a functional endothelium (Figure 4B), TRPA1 (Figure 4C), NOS (Figure 4D) and GC (Figure 4E).

### Role of K^+^-Channels

**FIGURE 5:**
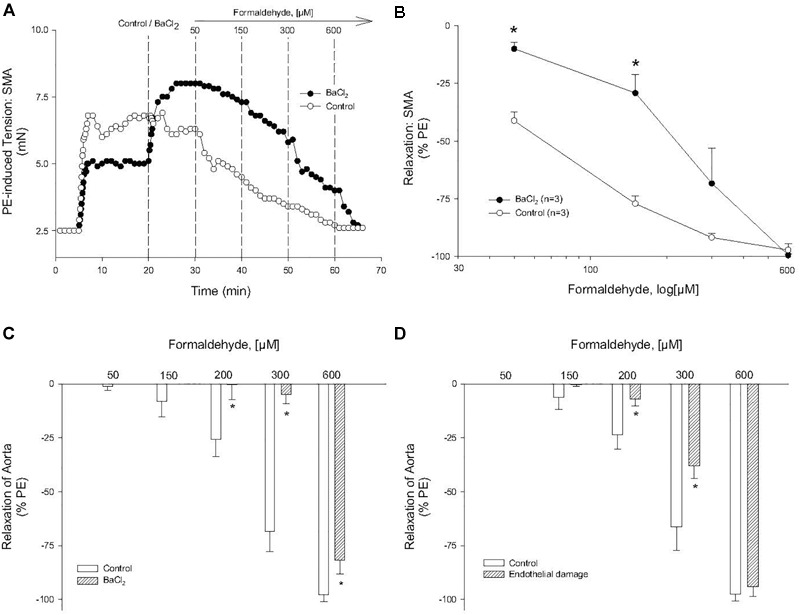
Role of potassium channel (K_IR_) in formaldehyde (FA)-induced relaxation in phenylephrine (PE) pre-contracted blood vessels. **(A)** Representative and **(B)** summary curves of FA-induced relaxation in SMA in the absence and presence of BaCl_2_. Summary curves of FA-induced relaxation in aorta in the absence and presence of **(C)** BaCl_2_ or **(D)** endothelium damage (ED). BaCl_2_ (1 mM) was added to PSS after stable PE-induced contraction and prior to cumulative addition of FA. ED was induced by 5 min of air perfusion. Values are means ± SE of 3–4 preparations. ^∗^, *P* < 0.05 vs. Control.

In SMA pre-contracted with 60K, FA induced an insensitive (rightward-shifted) relaxation (compared with both PE and U46,619). From these data, we surmised that alteration of smooth muscle membrane potential, likely due to K^+^ channel activation, contributed to FA-induced vasorelaxation in PE-contracted SMA. In confirmation, BaCl_2_ (1 mM), which blocks inward rectifier K^+^ channels, significantly inhibited FA-induced relaxation in PE-contracted SMA (Figure 5A,B) and also in aorta (Figure 5C). However, we observed no effect of the non-selective K^+^ channel inhibitor, TEA (tetraethylammonium, 10 mM), on FA-induced relaxation in SMA (Tables 3, 4). Collectively, these results indicate that the activation of K_IR_ channels but not of other K^+^ channels was involved in FA-induced vasorelaxation in PE-precontracted SMA. The endothelium also contributed to FA-induced relaxation in aorta (Figure 5D).

**Table 4:** The sensitivity of formaldehyde-induced vasorelaxation measured as the effective concentration inducing 50% relaxation (EC_50_; in μM) in isolated murine superior mesenteric artery (SMA; male) pre-contracted with PE in the absence and presence of functional endothelium or selective inhibitors of relaxation.

SMA	Control EC_50_	EC_50_
(+treatment)	(PE+vehicle)	(ED or PE+inhibitor)
+ED	61 ± 8	142 ± 14^∗^
+LNAME	52 ± 8	207 ± 10^∗^
+A967079	52 ± 6	95 ± 18^∗^
+ODQ	76 ± 15	182 ± 16^∗^
+BaCl_2_	63 ± 10	248 ± 56^∗^
+TEA	75 ± 3	93 ± 15

*Values = means ± SE. Abbr.: PE, phenylephrine, 10 μM; ED, endothelial dysfunction induced by mechanical air perfusion; LNAME, N^ω^-nitro-_*L*_-arginine methyl ester hydrochloride, 100 μM; A967079, TRPA1 antagonist, 3 μM; ODQ, 1 h-[1,2,4]oxadiazolo[4,3-a]quinoxalin-1-one, BaCl_*2*_, barium chloride, 1 mM; TEA, tetraethylammonium. *N* = 3–6 per group. ^∗^, *P* < 0.05 vs. Control (PE+vehicle).*

## Discussion

To our knowledge, this is the first study to propose that FA may contribute to postprandial hyperemia via a TRPA1- an endothelium-mediated SMA vasodilation. Because rodents feed at night and sleep (fast) during the day, we measured and observed a circadian difference in urinary formate levels indicating endogenous FA level likely increases with feeding. As far as we know, this is a novel observation. Furthermore, we show that SMA is sensitive to FA significantly more so than aorta, and that FA not its metabolite, formate, stimulates a robust and reversible vasorelaxation. The SMA is a major arterial blood supply to the GI tract and it dilates postprandially in response to many stimuli e.g., nutrients, vago-vagal reflex, NO, substance P, etc., (Chou and Coatney, 1994; Lucchini et al., 1996). Although FA is oft considered a toxicant, its natural abundance in food, water and beverages (Committee on Aldehydes, 1981; Conklin et al., 2011) and as an abundant metabolic product make it a plausible stimulus of GI blood flow. As this is the first suggestion of this physiological role, more research will be needed to establish FA as a *bona fide* contributor to postprandial hyperemia *in vivo*.

**FIGURE 6:**
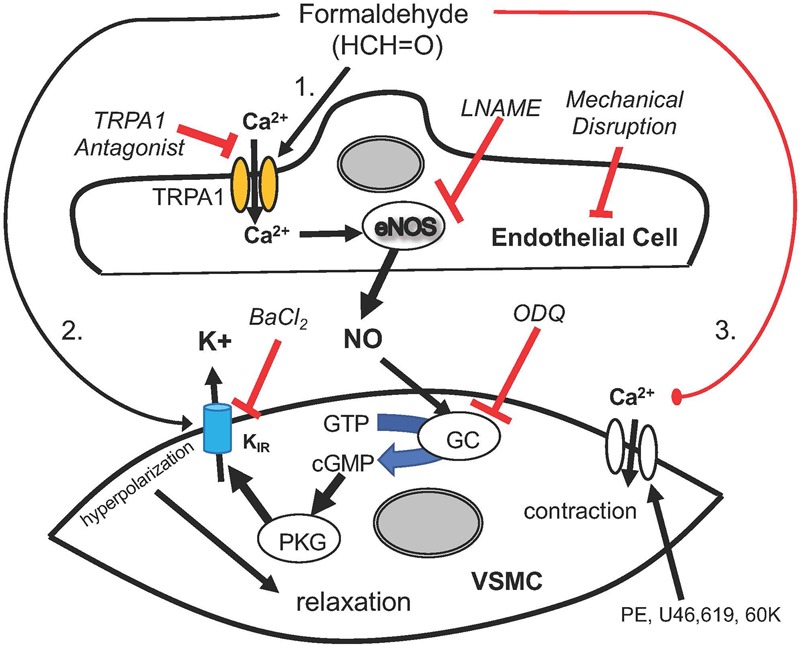
A cartoon depicting the mechanisms by which formaldehyde (FA) induces vasorelaxation in SMA precontracted by phenylephrine (PE), thromboxane A_2_ analog (U46,619) and 60 mM potassium solution (60K). FA appeared to induce a sensitive and sequential mechanism that begins with opening of Transient Receptor Potential Ankyrin 1 (TRPA1) cation channels, Ca^2+^ entry into endothelial cell, eNOS activation, NO formation, GC activation and cGMP formation with Protein Kinase G (PKG) mediated opening of K_IR_ channels leading to VSMC hyperpolarization and relaxation. Several of these steps were significantly impaired by a selective antagonist or treatment, including TRPA1 (A967079), eNOS (LNAME), endothelium (impaired by air perfusion), guanylyl cyclase (GC; ODQ) and K_IR_ channel (BaCl_2_). As demonstrated, this sequential pathway was sensitive to exogenous FA (30–150 μM) in the physiological range that was altered at least 2–3× during feeding. Other less sensitive yet equally efficacious pathways of vasorelaxation included VSMC hyperpolarization and a voltage-insensitive mechanism that reversed 60K-induced contraction implicating closure of VSMC Ca^2+^ channels.

We show that there are 3 distinct pathways of FA-induced vasorelaxation in SMA (Figure 6) and while this is fascinating in its own right, we focused on the mechanism of the most sensitive, and thus, most likely physiological pathway. FA-induced vasorelaxation requires a functional endothelium, TRPA1 channels, NOS (presumably eNOS), guanylyl cyclase (GC/cGMP) and K_IR_ channels. Likely, the TRPA1 and NOS components are in endothelial cells, whereas the latter two components are likely present in vascular smooth muscle cells (VSMC). From these collective data, we propose a model featuring a stepwise sequence of events that is quite typical of agonists that elicit EDRF/NO such as ACh (see model in Figure 6). That FA activates a TRPA1 channel likely present on the SMA endothelium is paralleled by FA action in rat aorta (Zhang et al., 2018) and by systemically administered propofol – a general anesthetic that induces TRPA1-dependent hypotension (Sinha et al., 2015). Similarly, TRPA1 is a known target of formalin (solution of FA and methanol) that increases blood flow and pain (McNamara et al., 2007). With regard to formalin, we show that it is likely the FA in formalin and not the methanol, which can be metabolized to FA, that elicits vasorelaxation.

TRPA1 is a well-known target of unsaturated aldehydes, such as acrolein and crotonaldehyde (Andre et al., 2008; Bessac et al., 2008; Conklin, 2016) and cinnamaldehyde (Pozsgai et al., 2010), but its activation by FA is somewhat surprising given the proposed mechanism of unsaturated aldehyde-mediated activation via conjugation with TRPA1 N-terminal free cysteines (Macpherson et al., 2007). In addition, several unsaturated aldehydes including acrolein, cinnamaldehyde and 4HNE induce sensitive and robust vasodilation in mesenteric arteries (Romero et al., 1997; Awe et al., 2006) and other blood vessels (Yanaga et al., 2006) and tracheal smooth muscle (Cheah et al., 2014) indicating perhaps a shared (universal) aldehyde-TRPA1 pathway.

In any case, TRPA1 is a cation (calcium) channel and a promiscuous receptor concentrated in sensory fibers (unmyelinated C-fibers) that mediate pain reception/ transmission (nociceptors), and it is also present in vasculature (Earley et al., 2009; Sinharoy et al., 2017). Activation of peripheral sensory fiber TRPA1 leads to pain signaling and release of vasoactive peptides, substance P (SubP) and cGRP (Trevisan et al., 2016), which may contribute to increased vasodilation. We confirm localization of TRPA1 in mouse DRG and show specific staining in the endothelium of murine SMA but not in aorta. Functionally, the specific TRPA1 antagonist, A967079 (IC_50_ = 289 nM), significantly blocks *the most sensitive component* of the FA-induced relaxation and shifts the EC_50_ to the right quantitatively similar to that of endothelium disruption but less so than LNAME treatment – further supporting a role of endothelial cell TRPA1. Above 150 μM FA – likely an *unphysiological* level of FA – the TRPA1 antagonist did not affect FA relaxation reflecting a non-TRPA1 pathway(s), yet perhaps via TRPV1 or TRPV4 channels (Ma et al., 2008). We cannot rule out the possibility that additional components in the vascular wall (perivascular nerves, SubP, CGRP, etc.) also may contribute to overall FA-induced relaxation (Nilius et al., 2012).

Formaldehyde-induced vasorelaxation is well-documented for over 4 decades, yet the complex mechanisms of this phenomenon are only now becoming clearer. In contrast to the idea that FA induces endothelial dysfunction (Yu and Deng, 1998), our data indicate that endothelial dysfunction itself likely would block only the physiological (sensitive) component of FA-induced vasorelaxation. We did not observe evidence of FA-induced endothelial dysfunction even after exposing SMA to 1,000 μM FA. Moreover, in isolated human CABG blood vessels with endothelial dysfunction, FA still induces relaxation but in a far less sensitive manner (EC_50_, 315 ± 0 μM) (Conklin et al., 2004) than that present in healthy mice (∼65 μM; this study).

It is clear that the vascular effects of FA *ex vivo* are not a result of FA metabolism into formate (primary metabolite). For example, formate addition (up to 2.1 mM) fails to evoke a strong vascular response, and thus, oxidative metabolism of FA regulates FA levels and subsequently any FA-evoked physiological vascular responses. Similarly, formate fails to recapitulate formalin-induced vasodepressor response in rats *in vivo* (Strubelt et al., 1990). Because FA metabolism involves a reduced glutathione (GSH)- and FALDH-dependent (aka ADH3) hemi-thioacetal formation (Kaiser et al., 1991), this process may be more affected by changes in oxidant load and GSH depletion in the vascular wall or in neurons, for example (Materazzi et al., 2012). As shown previously, deficiency in glutathione-dependent metabolism of acrolein (i.e., GSTP-null mice) increases susceptibility to acrolein-induced endothelial dysfunction *in vivo* and *in vitro* (Conklin et al., 2009). Whether or not FALDH deficiency or GSH depletion likewise alters vascular sensitivity to FA-induced relaxation (or toxicity) remains to be tested.

In conclusion, we describe a sensitive mechanism of FA-induced vasorelaxation that is sequentially dependent on an endothelium-localized TRPA1 receptor (EDRF/NO) and a VSMC GC/PKG/K_IR_ pathway in SMA. We hypothesize that this sensitive pathway is only a part of the postprandial hyperemia reflex that increases blood flow to the GI tract to augment digestion and nutrient absorption.

## Author Contributions

LJ and DC planned and conducted the experiments, analyzed and interpreted the data, and wrote the manuscript. LG, GJ, MN, PL, and MM generated and interpreted the data. MN provided expertise and reagents. MN, AB, and SS helped with the analysis, interpretation of the data, and contributed to the editing of the manuscript. DC was the guarantor of this work, had full access to all data and took responsibility for the integrity and accuracy of the data.

## Conflict of Interest Statement

The authors declare that the research was conducted in the absence of any commercial or financial relationships that could be construed as a potential conflict of interest.

## Supplementary Material

FIGURE S1 Regulators of agonist-induced tension in superior mesenteric artery (SMA). **(A)** Representative tracings of PE-induced tension and ACh-induced relaxation in SMA with intact and mechanically-impaired endothelium (ED). **(B)** Phenylephrine (PE), U46,619- and 60K-induced tension (mN) in SMA in the absence and presence of LNAME. **(C)** FA-induced relaxations (%) in PE, U46,619- and 60K-precontracted SMA in the absence and presence of LNAME. **(D)** FA-induced relaxations (converted to 100 %) in PE, U46,619- and 60K-precontracted SMA in the absence and presence of LNAME. E) Summary data of PE-induced tension in SMA in the absence and presence of LNAME, BaCl2, ED, and ODQ. Values are means ± SE of 3–4 preparations. ^∗^, *P* < 0.05 vs. Control.
