# Supplementary figures and images for: Formaldehyde Induces Mesenteric Artery Relaxation via a Sensitive Transient Receptor Potential Ankyrin-1 (TRPA1) and Endothelium-Dependent Mechanism: Potential Role in Postprandial Hyperemia
Source: Front Physiol. 2019 Mar 28;10:277. doi: 10.3389/fphys.2019.00277 (PMC6448550; doi:10.3389/fphys.2019.00277)

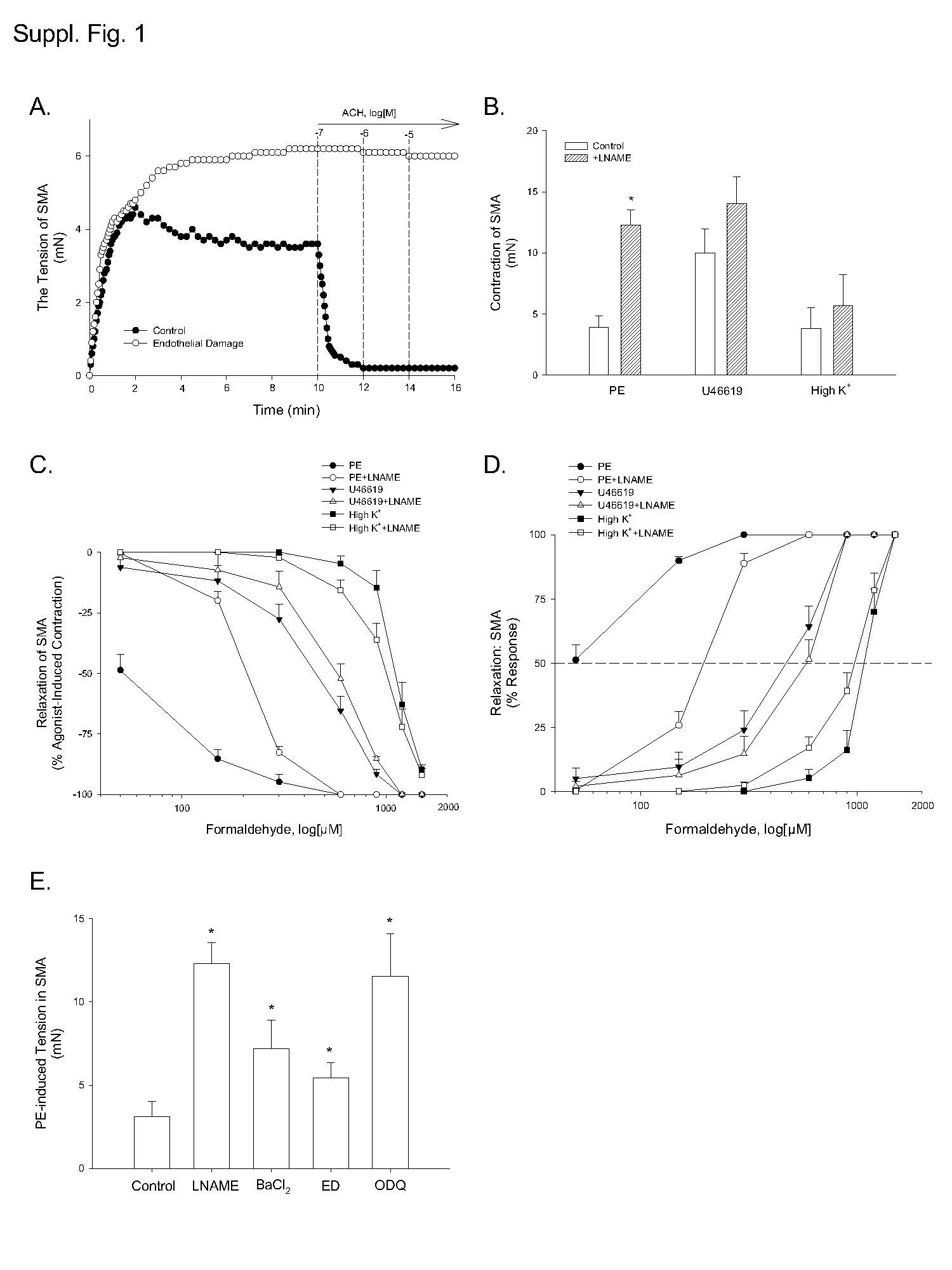

Supplement: FIGURE S1 — Regulators of agonist-induced tension in superior mesenteric artery (SMA). (A) Representative tracings of PE-induced tension and ACh-induced relaxation in SMA with intact and mechanically-impaired endothelium (ED). (B) Phenylephrine (PE), U46,619- and 60K-induced tension (mN) in SMA in the absence and presence of LNAME. (C) FA-induced relaxations (%) in PE, U46,619- and 60K-precontracted SMA in the absence and presence of LNAME. (D) FA-induced relaxations (converted to 100 %) in PE, U46,619- and 60K-precontracted SMA in the absence and presence of LNAME. E) Summary data of PE-induced tension in SMA in the absence and presence of LNAME, BaCl2, ED, and ODQ. Values are means ± SE of 3–4 preparations. ∗, P < 0.05 vs. Control. [file Image_1.JPEG]
